# Effect of Neonatal Azithromycin on All-Cause and Cause-Specific Infant Mortality: A Randomized Controlled Trial

**DOI:** 10.4269/ajtmh.22-0245

**Published:** 2022-11-07

**Authors:** Ali Sié, Mamadou Bountogo, Alphonse Zakane, Guillaume Compaoré, Thierry Ouedraogo, Elodie Lebas, Fanice Nyatigo, Huiyu Hu, Jessica Brogdon, Benjamin F. Arnold, Thomas M. Lietman, Catherine E. Oldenburg, for the NAITRE Study Team

**Affiliations:** ^1^ Centre de Recherche en Santé de Nouna, Nouna, Burkina Faso;; ^2^ Francis I Proctor Foundation, University of California, San Francisco, California;; ^3^ Department of Ophthalmology, University of California, San Francisco, California;; ^4^ Department of Epidemiology & Biostatistics, University of California, San Francisco, California

## Abstract

Mass azithromycin distribution reduces all-cause childhood mortality in some high-mortality settings in sub-Saharan Africa. Although the greatest benefits have been shown in children 1 to 5 months old living in areas with high mortality rates, no evidence of a benefit was found of neonatal azithromycin in a low-mortality setting on mortality at 6 months. We conducted a 1:1 randomized, placebo-controlled trial evaluating the effect of a single oral 20-mg/kg dose of azithromycin or matching placebo administered during the neonatal period on all-cause and cause-specific infant mortality at 12 months of age in five regions of Burkina Faso. Neonates were eligible if they were between the ages of 8 and 27 days and weighed at least 2,500 g at enrollment. Cause of death was determined via the WHO 2016 verbal autopsy tool. We compared all-cause and cause-specific mortality using binomial regression. Of 21,832 infants enrolled in the study, 116 died by 12 months of age. There was no significant difference in all-cause mortality between the azithromycin and placebo groups (azithromycin: 52 deaths, 0.5%; placebo, 64 deaths, 0.7%; hazard ratio, 0.81; 95% CI, 0.56–1.17; *P* = 0.30). There was no evidence of a difference in the distribution of causes of death (*P* = 0.40) and no significant difference in any specific cause of death between groups. Mortality rates were low at 12 months of age, and there was no evidence of an effect of neonatal azithromycin on all-cause or cause-specific mortality.

## INTRODUCTION

Mass azithromycin distribution to children age 1 to 59 months has been shown to reduce all-cause childhood mortality in some high-mortality settings in sub-Saharan Africa.^1^ In Niger, children who were treated between the ages of 1 and 5 months had an approximately 25% reduced risk of mortality 6 months later. This finding led to the hypothesis that early azithromycin treatment during infancy may offer the most protection against mortality. Previous azithromycin for childhood mortality trials have excluded children younger than 1 month of age because of concerns related to infantile hypertrophic pyloric stenosis (IHPS). The Neonates and Azithromycin: an Innovation in the Treatment of Children (NAITRE) trial was designed to evaluate the efficacy and safety of a single oral dose of azithromycin administered to neonates age 8 to 27 days for prevention of mortality.^2^ The primary outcome was 6 months of age, and the trial found no evidence of a difference in mortality in infants receiving azithromycin versus placebo at 6 months.^3^ The trial monitored children until 12 months of age to assess both infant and cause-specific mortality through 12 months of age. This design allowed for evaluation of any longer term effects of azithromycin, which may have additional statistical power beyond the primary outcome.

Azithromycin is a broad-spectrum antibiotic, and any effect on mortality presumably is via a reduction in infectious burden. Verbal autopsy data have suggested that azithromycin reduces mortality related to meningitis, dysentery, and malaria in children age 1 to 59 months.^4^ Most infant mortality after the first week of life is infectious.^5^ Here, we evaluated the effect of neonatal azithromycin administration on all-cause and cause-specific mortality at 12 months of age.

## METHODS

### Trial methods.

Complete methods for the trial have been reported previously.^2^^,^^3^ In brief, NAITRE was a 1:1 randomized, placebo-controlled trial evaluating whether a single, oral 20-mg/kg dose of azithromycin administered to infants between 8 and 27 days of age reduces all-cause infant mortality through 12 months of age. Participants were enrolled between April 2019 and December 2020, and follow-up occurred through December 2021. The study was reviewed and approved by the Comité d’Ethique pour la Recherche en Santé in Ouagadougou, Burkina Faso (Protocol No. 2018-10-123) and the Institutional Review Board at the University of California, San Francisco (Protocol No. 18-25027). Written informed consent was obtained from at least one parent/guardian of each enrolled infant. The trial was overseen by a Data and Safety Monitoring Committee that consisted of experts in pediatrics, pediatric infectious disease, biostatistics, and bioethics. A single interim analysis was conducted after follow-up data for approximately one third of the target sample size were available. The Data and Safety Monitoring Committee recommended continuation of the trial upon review of the interim analysis.^3^

### Study setting.

Participants were enrolled in primary health-care facilities in five regions of Burkina Faso. The trial was designed to assess both safety and efficacy of azithromycin. Because of concerns that neonatal azithromycin administration could lead to increased risk of IHPS,^6^ facilities were selected to be within 1 hour of a facility with diagnostic ultrasound capacity and within 4 hours of a hospital with pediatric surgical capacity.

### Participants.

Eligible neonates were between 8 and 27 days of age, weighed at least 2,500 g at the time of enrollment, were able to feed orally (to facilitate adherence to the study medication), and were planning to stay in the study area for the duration of the study period.

### Intervention.

Participants were randomized to a single oral dose of azithromycin (20 mg/kg) or equivalent volume of matching placebo. Participants were weighed at baseline using a standard infant scale, weights were entered into the study’s mobile data collection platform, and the tablet calculated the volume of study medication to administer to the child. All study treatments were observed directly by study staff. Azithromycin and matching placebo were donated by Pfizer, Inc. (New York, NY).

### Randomization, masking, and allocation concealment.

Participants were randomized in a 1:1 fashion without blocking or stratification. The randomization list was generated by the study’s unmasked data team and implemented by the study’s mobile data application. After enrollment and baseline procedures were complete, the mobile application indicated a letter to study staff. Four letters each (eight total) were assigned randomly to azithromycin and placebo, and the medication bottles were labeled with these letters. Medication bottle labels were identical with the exception of the letter. The study staff member treated the child with a medication bottle labeled with the letter corresponding to that shown in the mobile data application. Participants, study staff members, and investigators were masked to treatment assignment and did not know which letters corresponding to which treatment. Allocation concealment was achieved by not showing the study staff member the child’s randomized letter until after enrollment and baseline procedures were complete.

### Outcomes.

Vital status (died, alive, unknown) was assessed at every follow-up visit (21 days after enrollment and at 3, 6, and 12 months of age). Any child who died by the 12-month study visit was counted as having died. At each study visit, caregivers were interviewed about whether their child had been hospitalized or visited a health-care facility for a reason other than routine care (e.g., vaccinations or well-child visits). Caregivers were asked why they sought care for their child, including for malaria, pneumonia, diarrhea, or fever without another diagnosis. For the 12-month study visit, a window of 6 weeks before and after 12 months of age was prespecified for the timing of the visit.

### Safety monitoring.

Evaluation of safety end points was focused primary on the 3-month period after treatment. The intervention was a single oral dose of azithromycin, and we anticipated (based on previous data) that most adverse events would be gastrointestinal and occur within days to weeks after treatment.^7^ The primary serious adverse event of concern was IHPS, a rare but serious condition that requires surgical intervention. Observational studies previously suggested a link between early life macrolide exposure and IHPS.^6^ IHPS is a condition of early infancy, and thus active safety monitoring was designed specifically around the weeks and months immediately after treatment.^3^ As reported previously, a single case of IHPS was detected in the azithromycin arm. A supplemental questionnaire was developed for the verbal autopsy to evaluate whether the circumstances surrounding each child’s death could potentially be related to IHSP. Caregivers were also instructed to contact study staff with any other concerns related to their child’s participation in the study.

### Cause-of-death assessment.

Cause of death was determined using the WHO 2016 verbal autopsy instrument.^8^ Interviews were conducted with caregivers of deceased participants approximately 3 months after the date of death, in accordance with local standards for interviews following the mourning period. Data were collected electronically on the study’s mobile data application. Cause of death was assigned via InSilicoVA in R (R Foundation for Statistical Computing, Vienna, Austria), which uses an automated algorithm to assign causes of death and is compliant with the 2016 WHO verbal autopsy instrument.^9^ We considered the most likely cause of death as assigned by the algorithm as the cause of death.

### Sample size considerations.

The sample size for the trial was based on the primary end point: mortality at 6 months of age. We assumed a mortality probability of 35 per 1,000 live births and loss to follow-up of 10%. Under these assumptions, a sample size of 10,856 per arm (*N* = 21,712 total) was estimated to yield at least 80% power to detect a 20% decrease in mortality at 6 months. The observed mortality rate at 6 months was substantially less than estimated (0.44% in the azithromycin arm and 0.52% in the placebo arm).^3^ If the 6-month mortality rate observed in the trial was preserved, a sample size of nearly 250,000 infants would have been required to achieve 80% power.

### Statistical methods.

All analyses considered a two-sided alpha of 0.05 statistically significant and were conducted in R (R Foundation for Statistical Computing). All-cause and cause-specific mortality were analyzed using binomial regression with a complementary log-log link, with treatment group as the sole predictor to estimate the relative hazard of mortality between treatment groups. Among children who died, we evaluated the distribution of causes of death between arms using Fisher’s exact test. Other outcomes, including hospitalization and/or death, hospitalization, and health-care use, were analyzed similarly to the mortality end points. For mortality end points, the main analyses were restricted only to children who were measured in the prespecified visit window (12 months of age ± 6 weeks). As a sensitivity analysis, we included all children with known vital status regardless of whether the visit fell within the prespecified window.

## RESULTS

**Figure 1.**
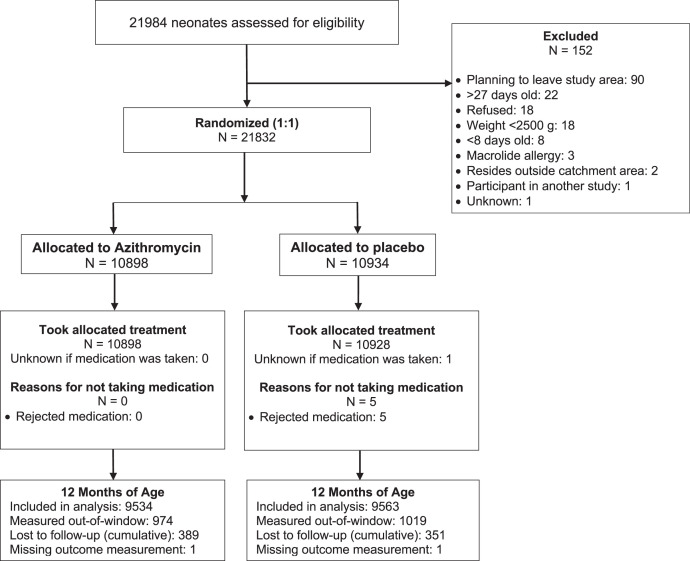
CONSORT flow diagram of participant enrollment, randomization, and follow-up.

Of 21,832 infants enrolled in the study, 10,898 were randomized to azithromycin and 10,928 to placebo (Figure 1). Infants were a median of 11 days old at enrollment and 50% were female (Table 1). At 12 months, 19,097 infants (87%) had undergone vital status measurements during the prespecified window and were included in the main analyses, and an additional 1,993 had vital status measurements outside the window (96% overall with known vital status at 12 months of age). Of the 19,097 infants included in the main analyses, 9,534 (87%) were in the azithromycin group and 9,563 (87%) were in the placebo group. In a sensitivity analysis including all children regardless of whether their 12-month visit fell within the prespecified window, 10,508 (96%) were in the azithromycin group and 10,582 (97%) were in the placebo group. Baseline characteristics of children who were and were not retained at 12 months were broadly similar Supplemental Table 1).

**Table 1:** Baseline characteristics by randomized treatment group

Characteristic	Azithromycin (*n* = 10,898)	Placebo (*n* = 10,934)	Overall (*N* = 2,1832)
Age, days; median (IQR)	11 (9–15)	11 (9–14)	11 (9–14)
Gender, *n *(%)			
Female	5,413 (49.7)	5,431 (49.7)	10,844 (49.7)
Male	5,485 (50.3)	5,503 (50.3)	10,988 (50.3)
Region, *n *(%)			
Center	919 (8.4)	951 (8.7)	1,870 (8.6)
Boucle du Mouhoun	1,299 (11.9)	1,329 (12.2)	2,628 (12.0)
Cascade	2,009 (18.4)	1,977 (18.1)	3,986 (18.3)
Center Ouest	1,217 (11.2)	1,211 (11.1)	2,428 (11.1)
Hauts-Bassins	5,454 (50.0%)	5,465 (50.0)	10,919 (50.0)
Birthweight, g; median (IQR)	3,000 (2,700–3,250)	3,000 (2,700–3,260)	3,000 (2,700–3,250)
Pregnancy type, *n *(%)			
Singleton	10,702 (98.2)	10,753 (98.3)	21,455 (98.3)
Multiple	195 (1.8)	177 (1.6)	372 (1.7)
Mother’s age, years; median (IQR)	25 (21–30)	25 (21–30)	25 (21–30)
Mother’s education, *n *(%)			
None	5,910 (54.2)	6,029 (55.1)	11,939 (54.7)
Primary	1,990 (18.3)	1,978 (18.1)	3,968 (18.2)
Secondary or above	2,997 (27.5)	2,923 (26.7)	5,920 (27.1)
No. of antenatal visits, median (IQR)	4 (3–5)	4 (3–5)	4 (3–5)

IQR = interquartile range.

By 12 months of age, 116 participants (0.6%) had died, of whom 52 (0.5%) were in the azithromycin group and 64 (0.7%) were in the placebo group. This corresponded to a 19% relative reduction in mortality in infants receiving azithromycin compared with placebo (hazards ratio [HR], 0.81; 95% CI, 0.56–1.17; *P* = 0.30; Table 2). No additional deaths were identified when including all children regardless of the visit window; sensitivity analysis including the additional children did not change results Supplemental Table 2). There was no evidence of a difference in any other clinical outcome, including hospitalization or clinic visits (Table 2).

**Table 2:** Mortality, hospitalizations, and clinic visits through 12 months of age

Variable	Azithromycin			Placebo			Hazard ratio (95% CI)	*P* value*
	*N*	*n*	%	*N*	*n*	%		
12-Month mortality	9,534	52	0.5	9,563	64	0.7	0.81 (0.56–1.17)	0.30
Death and/or hospitalization	9,542	222	2.3	9,571	236	2.5	0.94 (0.97–1.13)	0.51
Hospitalization	9,494	172	1.8	9,512	173	1.8	1.00 (0.81–1.23)	0.96
Any clinic visit	9,634	4,586	47.6	9,643	4,565	47.3	1.01 (0.97–1.05)	0.72
Cause-specific clinic visits†								
Malaria	9,634	1,145	11.9	9,643	1,104	11.4	1.04 (0.96–1.13)	0.34
Pneumonia	9,634	2,256	23.4	9,643	2,201	22.8	1.03 (0.97–1.09)	0.33
Diarrhea	9,634	1,559	16.2	9,643	1,507	15.6	1.04 (0.97–1.11)	0.30
Fever‡	9,634	2,205	22.9	9,643	2,201	22.8	1.00 (0.94–1.06)	0.91

* Permutation *P* value (10,000 replications).

† Data and classifications of illness from caregiver-reported survey.

‡ Fever in the absence of another diagnosis.

Verbal autopsy results were available for 112 (97%) of the 116 deaths. The most common causes of death were malaria (*n* = 38, 34%), pneumonia and other acute respiratory tract infections (*n* = 28, 25%), other unspecified infectious diseases (*n* = 21, 19%), neonatal sepsis (*n* = 9, 8%), diarrheal disease (*n* = 5, 4%), acute abdomen (*n* = 5, 4%), accident (*n* = 3, 3%), severe malnutrition (*n* = 2, 2%), and HIV-related death (*n* = 1, 1%). Infants whose death was determined to be the result of malaria died at an average age of 4.7 months (SD, 2.9 months) compared with 3.3 months (SD, 2.3 months) for pneumonia and 20 days (SD, 4.6 days) for neonatal sepsis Supplemental Table 3). Among children who died, there was no evidence of a difference in the distribution of causes of death as determined by verbal autopsy by randomized treatment group (Fisher’s exact test, *P* = 0.40). No children had symptoms suggestive of IHPS prior to their death.

There was no evidence of a difference in any specific cause of mortality between treatment groups. Infants randomized to azithromycin were less likely to die of malaria compared with placebo (HR, 0.52; 95% CI, 0.26–1.00; Table 3). Deaths attributed to neonatal sepsis were more common in the azithromycin group compared with placebo group (HR, 3.52; 95% CI, 0.85–23.7). Other causes of death were similar between treatment groups (Table 3).

**Table 3:** Causes of mortality* through 12 months of age

Cause of morality	Azithromycin			Placebo			Hazard ratio (95% CI)
	*N*	*n*	%	*N*	*n*	%	
Malaria	10,511	13	0.12	10,583	25	0.24	0.52 (0.26–1.00)
Acute respiratory infection	10,511	13	0.12	10,583	15	0.14	0.87 (0.41–1.84)
Other infectious cause	10,511	11	0.10	10,583	10	0.09	1.11 (0.47–2.66)
Neonatal sepsis	10,511	7	0.07	10,583	2	0.02	3.52 (0.85–23.67)
Diarrheal disease	10,511	3	0.03	10,583	2	0.02	1.51 (0.25–11.47)
Acute abdomen	10,511	2	0.02	10,583	3	0.03	0.67 (0.09–4.05)
Severe acute malnutrition	10,511	1	0.01	10,583	1	0.01	1.01 (0.04–25.46)
Accident	10,511	1	0.01	10,583	2	0.02	0.50 (0.02–5.26)
HIV/AIDS	10,511	1	0.01	10,583	0	0	NA

NA = not applicable.

* Classifications of cause of death estimated using the InSilicoVA algorithm.

## DISCUSSION

In this study of azithromycin versus placebo in early infancy for prevention of infant mortality, we were unable to demonstrate a difference in all-cause mortality by 12 months of age in infants receiving azithromycin compared with placebo. Few infants died between 6 and 12 months of age in this cohort (24 additional deaths) despite low loss to follow-up, suggesting that the study population was at low risk of mortality. The study was designed both for safety and efficacy, and particular attention was given to safety related to the potential for IHPS after early macrolide exposure. Because of concerns that smaller babies may be at increased risk of IHPS,^10^ babies weighing < 2,500 g at enrollment were excluded from the trial. Low birthweight and underweight neonates are at the highest risk of mortality, and thus exclusion of this vulnerable population may have led to lower mortality rates. In addition, enrolling facilities were chosen for their proximity to facilities with diagnostic ultrasound capacity and tertiary hospitals with pediatric surgery capacity. This likely selected for greater socioeconomic status and more urban settings with lower mortality rates. The overall point estimate at 12 months of age (19% relative reduction in the azithromycin group) was similar to the point estimate from the trial’s primary end point (15% relative reduction at 6 months of age), but the CIs were wide as a result of the low event rate.^3^

As expected, causes of death were mostly infectious. Although the overall distribution of causes of death did not differ between arms, deaths determined to be the result of malaria were fewer in children randomized to azithromycin compared with placebo. This finding is consistent with the MORDOR study,^4^ which documented a 22% reduced risk of malaria mortality in communities receiving azithromycin versus placebo. Azithromycin has mild in vitro antimalarial properties by targeting the plasmodial apicoplast,^11^ but trials have found mixed results of azithromycin on malaria parasitemia.^12^^–^^15^ Verbal autopsy determination of malaria mortality is prone to misclassification.^16^^–^^19^ For example, verbal autopsies frequently classify febrile illness without another known cause as malaria.^20^ Deaths classified as malaria per verbal autopsy may have been the result of other infectious causes for which azithromycin may have greater efficacy. As expected, deaths coded as malaria occurred later than other etiologies. In rural Burkina Faso, facility-diagnosed malaria cases have been shown to peak at 7 to 12 months of age.^21^ The number of malaria deaths was small, and the CI included 1, and this finding may have been a result of chance.

Although rare, neonatal sepsis-related deaths were more common in infants receiving azithromycin compared with placebo, although this difference was not statistically significant and CIs were very wide. The primary analysis found an increased risk of serious adverse events within 28 days of treatment in neonates receiving azithromycin compared with placebo, including both death and hospitalization, although the absolute number of serious adverse events was very small.^3^ All deaths before 28 days of age were coded as neonatal sepsis or were the result of an accident. That there were more neonatal sepsis deaths in the azithromycin group does not necessarily provide insight into mechanism behind the difference in serious adverse events, and the small numbers may mean any differences are the result of chance. However, if presumptive antibiotic treatment selects for resistant infections, it is possible that these cases could be more difficult to treat. Macrolide use may co-select for beta-lactam resistance, and amoxicillin is commonly first-line therapy in the study area.^22^^,^^23^ However, the number of deaths within several weeks of treatment was very small and no firm conclusions can be drawn.

The results of this study must be considered in light of several limitations. Despite the overall large sample size, the mortality rate was persistently low in this study, and cause-specific mortality rates as a result were even lower. This limited statistical power and led to uncertainty in estimates. Although some results may be interpreted as hypothesis-generating, trials in a higher mortality rate population would have to be conducted to confirm any results. The percentage of children who were lost to follow-up was greater than the percentage who died. If children who were lost to follow-up were more likely to die, our mortality rates could be underestimated and, if differential by arm, missingness could introduce bias into the estimates, which could obscure differences in mortality rates between arms. Because loss to follow-up was nondifferential by arm, bias in the estimate hazards ratio is unlikely. Causes of death were determined via verbal autopsy, which as discussed previously can be subject to misclassification. Verbal autopsies were completed a minimum of 3 months after the death of the child to respect the mourning period. It is possible that some circumstances related to the death were misremembered. Last, this study may not be generalizable to settings with higher mortality rates or different distributions of causes of death.

In this longer term follow-up of a trial of neonatal azithromycin treatment of prevention of infant mortality, we were unable to demonstrate a difference in all-cause or cause-specific mortality by 12 months of age. The mortality rate stayed persistently low across the study period, limiting statistical power. Although malaria deaths were less in the azithromycin group compared with placebo, the CI included 1. These results do not support a longer term benefit of azithromycin for the prevention of infant mortality, and add to the evidence against mass drug administration with azithromycin in low-mortality populations. Future studies could consider azithromycin interventions in higher mortality settings to understand whether there is a role for this intervention for preventing infant mortality in other settings.

## Supplemental files


Supplemental materials
