# Supplementary material for: Effect of Neonatal Azithromycin on All-Cause and Cause-Specific Infant Mortality: A Randomized Controlled Trial
Source: Am J Trop Med Hyg. 2022 Nov 7;107(6):1331–6. doi: 10.4269/ajtmh.22-0245 (PMC9768279; doi:10.4269/ajtmh.22-0245)
Supplement: Supplementary file 1 [file tpmd220245.SD1.pdf]

Supplemental Table 1. Baseline characteristics among children who were and were not included in the 12-month mortality outcome analysis

	Included in analysis (N=19097)	Excluded from analysis (N=2733)
**Age (days)**		
Median (Q1 to Q3)	11.0 (9.00 to 14.0)	11.0 (9.00 to 16.0)
Missing	0 (0%)	1 (0.0%)
**Sex**		
Female	9455 (49.5%)	1389 (50.8%)
Male	9642 (50.5%)	1344 (49.2%)
**Region**		
Centre	1837 (9.6%)	33 (1.2%)
Boucle du Mouhoun	2031 (10.6%)	597 (21.8%)
Cascade	3490 (18.3%)	495 (18.1%)
Centre Ouest	2184 (11.4%)	244 (8.9%)
Hauts-Bassins	9555 (50.0%)	1363 (49.9%)
Missing	0 (0%)	1 (0.0%)
**Birthweight (g)**		
Median (Q1 to Q3)	3000 (2700 to 3270)	3000 (2700 to 3200)
Missing	471 (2.5%)	40 (1.5%)
**Pregnancy type**		
Singleton	18765 (98.3%)	2688 (98.4%)
Multiple	328 (1.7%)	44 (1.6%)
Missing	4 (0.0%)	1 (0.0%)
**Mother's age**		
Median (Q1 to Q3)	25.0 (21.0 to 30.0)	25.0 (20.0 to 30.0)
Missing	5 (0.0%)	1 (0.0%)
**Mother's education**		
None	10383 (54.4%)	1555 (56.9%)
Primary	3502 (18.3%)	465 (17.0%)
Secondary or above	5208 (27.3%)	712 (26.1%)
Missing	4 (0.0%)	1 (0.0%)
**No. of antenatal visits**		
Median (Q1 to Q3)	4.00 (3.00 to 5.00)	4.00 (3.00 to 5.00)
Missing	31 (0.2%)	3 (0.1%)

Supplemental Table 2. Mortality among all children with any vital status measurement regardless of timing of assessment

	Azithromycin			Placebo				
**12-month mortality**	**N**	**n**	**%**	**N**	**n**	**%**	**HR (95% CI)**	**P-value***
	10508	52	0.5%	10582	64	0.7%	0.82 (0.57 to 1.18)	0.40

Abbreviations: HR, hazards ratio; CI, confidence interval
*Permutation P-value (10,000 replications)
**Fever in the absence of another diagnosis

Supplemental Table 3. Average age of death in days by cause of death as determined by verbal autopsy

Cause of death	N	Mean age at death, days (standard deviation)
Malaria	38	157 (85)
Acute respiratory infection	28	115 (71)
Other infectious cause	21	116 (79)
Neonatal sepsis	9	20 (5)
Diarrheal disease	5	121 (61)
Acute abdomen	5	89 (35)
Severe acute malnutrition	2	226 (151)
Accident	3	40 (21)
HIV/AIDS	1	66 (NA)
